# Environment-Aware Proactive Beam Prediction in mmWave V2I via Multi-Modal Prior Mask Map

**DOI:** 10.3390/s26082488

**Published:** 2026-04-17

**Authors:** Changpeng Zhou, Youyun Xu

**Affiliations:** 1 School of Communications and Information Engineering, Nanjing University of Posts and Telecommunications, Nanjing 210003, China; 2022010310@njupt.edu.cn; 2 National Engineering Research Center of Communications and Networking, Nanjing University of Posts and Telecommunications, Nanjing 210003, China

**Keywords:** mmWave V2I communications, environment-aware, beam prediction, multi-model prior mask map, multi-sensor fusion

## Abstract

In millimeter wave V2I communication systems, accurate beam prediction is crucial for optimizing network performance and improving signal transmission efficiency. Traditional beam prediction methods mainly rely on single-modal data, which often fails to capture the comprehensive environmental information required for high accuracy prediction. In contrast, multi-modal approaches leverage complementary information from different data sources and offer a more promising solution. However, many existing fusion methods primarily depend on real-time sensory inputs and do not fully exploit stable environmental features in V2I scenarios, limiting the effective use of each modality. To address these limitations, this paper proposes a environment-aware proactive beam prediction method based on a multi-modal prior mask map (MMPMM), which integrates offline mapping with an online beam prediction network. Specifically, the method fuses information from images, point clouds, positions, and the MMPMM to predict the optimal beam index. The MMPMM provides channel-related prior information by extracting static V2I scene features offline without incurring any additional online measurement overhead. Experimental results on real-world datasets demonstrate that the proposed method achieves a Top-3 beam prediction accuracy of up to 71.23% while maintaining stable performance under the evaluated dynamic and degraded conditions, demonstrating its effectiveness in the considered scenarios.

## 1. Introduction

With the rapid development of intelligent transportation systems (ITS), vehicle to everything (V2X) communication has become a cornerstone for achieving intelligent mobility and autonomous driving [1]. By enabling real-time information exchange between vehicles and roadside infrastructures, V2X supports critical functions such as traffic flow optimization, safety coordination, and cooperative driving decisions [2,3]. Among various V2X paradigms, vehicle-to-infrastructure (V2I) communication plays a central role in collect traffic information and enabling edge intelligence for roadside decision [4,5]. To support these safety-critical and data-intensive services, wireless communication systems must achieve high data rates, ultra-low latency, and strong reliability. Due to its abundant spectrum resources and very high transmission capacity, millimeter wave (mmWave) communication has become a promising solution for meeting the stringent bandwidth and latency requirements of next-generation vehicular networks [6,7,8]. In addition to high-frequency mmWave communication technologies, vehicular networks have also been investigated from several complementary perspectives, including antenna design for vehicular communication systems, low-power wide-area networking solutions, and IoT-based sensing and data fusion frameworks [9,10,11,12]. These studies mainly address system-level design and alternative communication paradigms, providing a broader context for V2X development. However, they do not specifically address the unique challenges of high-frequency directional communications, which remain the focus of this work.

Nevertheless, applying mmWave to V2I scenarios faces significant challenges. Due to its high path loss, strong directionality, and susceptibility to blockage, mmWave links are highly sensitive to environmental changes [13]. In dynamic vehicular environments, frequent beam misalignment caused by vehicle mobility and occlusion can lead to link degradation or disconnection, which undermines the performance of latency sensitive applications [14,15,16]. Consequently, beam management-based massive multi-input–multi-output (mMIMO) becomes a critical factor for maintaining link stability, where efficient and accurate beam prediction techniques are essential. However, realizing robust and low-overhead beamforming under high mobility remains a fundamental challenge in mmWave vehicular networks.

Recent research has explored various beam management strategies. Conventional exhaustive beam training can guarantee optimal beam alignment, but it introduces heavy pilot signal overhead, which makes it unsuitable for real-time vehicular communication [14,17]. In [18,19,20], learning-based beam prediction approaches have been proposed to reduce overhead by exploiting channel state information (CSI). However, these methods typically rely only on communication data, ignoring the spatial and semantic dependencies between the environment and propagation conditions. When the surrounding environment changes, such as with line-of-sight (LOS) blockage, road layout variations, or traffic flow fluctuations, models without environmental awareness cannot anticipate these changes and fail to maintain stable performance.

In fact, environmental sensing data including RGB images, LiDAR point clouds, and GPS position provide rich semantic and spatial information that strongly correlates with wireless propagation characteristics. Image-assisted beam prediction leverages RGB images captured by BS mounted cameras to extract semantic information about vehicles, pedestrians, and obstacles [21,22,23,24,25,26,27]. The authors of [21] proposed one of the first vision-assisted communication frameworks. They used RGB images to recognize moving targets and obstacles, enabling proactive blockage prediction. The authors of [22] extended this idea by predicting the expected blockage duration through regression modeling, improving the system’s handover decisions. The authors of [23] combined visual sensing with deep reinforcement learning and used attention mechanisms to enhance feature representation. These studies show that visual information can effectively reduce beam search overhead. However, their performance is often affected by lighting, weather, and camera angle changes. Only vision models also lack accurate depth information, making them less reliable in complex or dynamic traffic environments.

LiDAR-based methods provide more precise geometric descriptions of the surrounding environment. LiDAR captures accurate 3D information, which helps model spatial structures and depth variations. The authors of [28] proposed a LiDAR-assisted federated learning framework, allowing vehicles to share model parameters without exchanging raw data, improving privacy and efficiency. The authors of [29] tested a LiDAR-assisted beam prediction model on a real-world large-scale dataset. LiDAR provides strong geometric priors for beam prediction, but it has limitations such as limited field of view and reduced performance in fog or rain. In dense urban environments, LiDAR signals may also be unstable when reflecting from moving targets.

GPS-assisted methods aim to predict beams using only location and trajectory information. They build a mapping between position sequences and beam index, realizing fast tracking with low computational cost. The authors of [30] proposed a GPS-assisted deep learning model for UAV communications. The authors of [31] used an enhanced convolutional neural network (CNN) trained on the real-world DeepSense 6G dataset. Their model improved beam prediction accuracy across multiple scenarios and reduced power loss. But GPS lacks environmental context, which limits its usefulness in non-line-of-sight (NLOS) conditions.

To overcome the limits of single-modality methods, researchers have recently turned to multi-modal fusion approaches. The authors of [32] proposed a multi-modal beam tracking method that combines 3D LiDAR and GPS data. Their model uses a multi-branch structure to jointly represent spatial geometry and trajectory information, enabling stable beam tracking between vehicles. The authors of [33] introduced a quantum Transformer network (QTN) for integrated sensing and communication (ISAC). By using quantum embeddings and attention mechanisms, the model captures correlations between different sensing modalities in distributed and semantic sensing. The authors of [34] proposed the environmental semantic communication (ESC) framework. Multiple cameras extract scene semantics at the edge and send only key features, instead of full images, to the BS. This approach reduces communication overhead and enhances environmental awareness. The authors of [35] introduced a Transformer-based multi-modal beamforming framework. It integrates localization clustering with multi-modal inputs to maintain communication quality under dynamic environmental changes.

Among the existing studies, the works most closely related to ours include semantic-assisted approaches based on perception outputs, as represented by [24,34], as well as recent Transformer-based multi-modal beam prediction methods, as represented by [26,34,36]. These methods leverage visual, LiDAR, or multi-modal sensing to incorporate environmental information into beam prediction. However, they predominantly rely on implicit feature fusion and do not explicitly model environment-related prior knowledge. In these approaches, environmental information is typically extracted via image segmentation, object detection, or multi-modal representation learning, and then introduced into the prediction model as auxiliary features. Despite improving perception capability, such designs remain essentially data-driven and lack an explicit structural representation of the environment.

As a consequence, the underlying relationship between environmental structures and wireless propagation is not explicitly captured. This limitation becomes critical in V2I scenarios, where static objects such as buildings and road infrastructure persist over long timescales and continuously shape reflection and blockage patterns in mmWave channels. Existing methods fail to effectively exploit such stable spatial characteristics. Moreover, directly utilizing raw sensing data inevitably introduces irrelevant information unrelated to propagation, which can degrade both efficiency and robustness. In addition, a comparison table with representative related works has been included to explicitly highlight the differences in modality usage, prior modeling, and prediction strategy, as shown in Table 1.

In contrast, this work adopts an environmental prior modeling paradigm, where environmental information is represented as structured prior knowledge rather than auxiliary inputs. In this paper, a multi-modal prior mask map (MMPMM) is constructed to explicitly encode propagation-relevant environmental structures. The resulting prior is incorporated as spatial guidance in beam prediction, enabling the model to exploit long-term environmental stability and facilitating a shift from reactive inference to proactive beam prediction.

Compared with existing environment-aware beam prediction methods, the proposed approach introduces an explicit prior to constrain the candidate beam space instead of relying solely on implicit multi-modal fusion. In contrast to semantic radio map-based approaches, it does not learn an explicit mapping from environmental semantics to channel or beam characteristics but instead leverages structured prior information to guide beam selection. Furthermore, unlike digital twin-based methods that require accurate environment reconstruction and continuous updates, the proposed MMPMM is constructed offline and introduces no additional computational overhead during online inference, resulting in a lightweight yet effective framework.

Specifically, the main contributions of this paper are threefold:

(1) We propose an environment-aware proactive beam prediction framework that combines multi-modal sensing with prior knowledge of the V2I scene. The framework uses RGB images and LiDAR data collected at the BS to build an offline prior mask map, which captures stable structures that affect mmWave propagation. During online beam prediction, the prior mask map is used together with real-time multi-modal sensor inputs to guide the selection of the optimal beam. In this process, the prior mask map provides spatial information that supports more reliable beam decisions under dynamic and uncertain conditions.
(2) We design a multi-modal prediction network composed of a feature extract module, a multi-modal fusion module, and a decision module. First, RGB images, LiDAR point clouds, GPS positions, and the prior mask map are processed by lightweight ResNet encoders to extract spatial features. These features are then passed through Transformer layers, which capture the link between environmental information and beam directions. Finally, a multilayer perceptron (MLP) predicts the optimal beam index from the fused representation.
(3) We validate the proposed method on the real-world DeepSense 6G dataset. The results show that it achieves higher accuracy in beam index prediction and realizing stronger received power, which demonstrates the advantage of environment-aware proactive beam prediction utilizing the MMPMM. The method also maintains stable performance in complex and dynamic scenes, demonstrating its practical value for V2I mmWave systems.

The remainder of this paper is organized as follows. Section 2 presents the system model and problem formulation. Section 3 introduces the design of the multi-modal prior mask map. Section 4 describes the proposed mmWave V2I proactive beam prediction method based on the multi-modal prior mask map. Section 5 reports the simulation results and performance evaluation. Section 6 concludes this paper.

Notation: Bold uppercase letters denote matrices, bold lowercase letters denote vectors, and lowercase letters denote scalars. $C^{M\times N}$ and $R^{M\times N}$ denote the spaces of M×N complex and real matrices, respectively. $A^{T}$ and $A^{H}$ denote the transpose and conjugate transpose of the matrix A. A[i,j] is the element of the *i*th row and the *j*th column in A; A[i,:] and A[:,j] are the *i*th row and the *j*th column of A, respectively. E{·} is the expectation operator.

## 2. System Model and Problem Formulation

**Figure 1:**
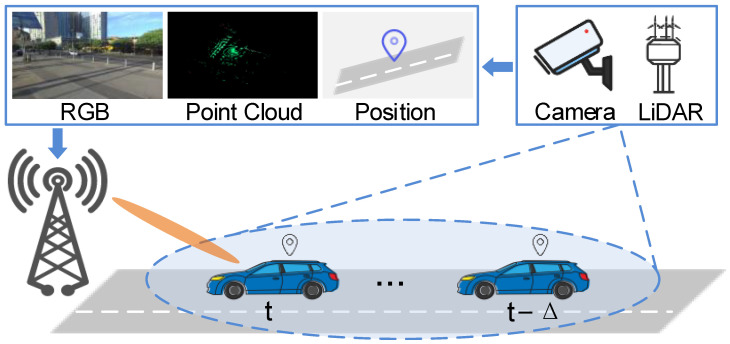
The V2I communication scenario for the multi-modal sensor data-based proactive beam prediction methods.

We consider a mmWave V2I communication system, where a roadside fixed BS equipped with multiple sensors and a uniform linear array (ULA) serves a vehicle user equipment (UE) moving along a road, as shown in Figure 1. The BS is mounted with RGB cameras and LiDAR sensors, which continuously perceive the surrounding environment, while UE is equipped with GPS for spatial synchronization.

### 2.1. System Model

The communication system adopts orthogonal frequency division multiplexing (OFDM) with *K* subcarriers. For simplicity, the BS is equipped with $N_{\mathrm{BS}}$ elements mmWave antenna array and communicates with a single antenna UE. The BS is assumed to employ an analog-only beamforming architecture [39], the pre-defined beam codebook is $F={\left\{f_{b}\right\}}_{b=1}^{B}$, $f_{b}\in C^{N_{\mathrm{BS}}\times 1}$ and *B* is the total number of beamforming vectors in the codebook. The received downlink signal at the UE for the *k*th subcarrier is given by

(1) $$y_{k}=H_{k}^{T}f_{b}s_{k}+n_{k},$$

where $H_{k}\in C^{N_{\mathrm{BS}}\times 1}$ is the channel matrix for the *k*th subcarrier, $s_{k}\in C$ is a transmitted symbol with $E\left\{|s_{k}{|}^{2}\right\}=P_{k}$, $P_{k}$ denoting the power budget per symbol, and $n_{k}$ is a noise from Gaussian distribution $n_{k}∼N_{C}\left(0,\sigma^{2}\right)$.

This study uses the geometric mmWave channel model [40]. The channel matrix at the *k*th subcarrier is

(2) $$H_{k}=\sum_{d=0}^{D-1}\sum_{l=1}^{L}\alpha_{l}e^{-j\frac{2\pi k}{K}d}g\left(dT_{s}-\tau_{l}\right)a(\theta_{l},\phi_{l}),$$

where *D* denotes the cyclic prefix length, *L* is the number of channel paths, $\alpha_{l}$, $T_{s}$, $\tau_{l}$, $\theta_{l}$, $\phi_{l}$ are the path gains, the sampling time, delay, path’s azimuth and elevation angles of the departure (AOD) of path *l*, respectively, and g(·) denotes the pulse shaping filter.

### 2.2. Problem Formulation

To achieve environment-aware proactive beam prediction in V2I, we leverage roadside multi-modal sensor resources and propose a MMPMM. This map is a structured environmental prior mask constructed at the BS using RGB images and LiDAR data. Formally, let $M_{\mathrm{BS}}=\left\{I^{\mathrm{RGB}},P^{\mathrm{LiDAR}}\right\}$ denote the multi-modal sensor data collected by the BS. The MMPMM is generated through a fusion function

(3) $$K=f_{\mathrm{mask}}\{M_{\mathrm{BS}}\},$$

where $f_{\mathrm{mask}}\left(\cdot \right)$ offline integrates image $I^{\mathrm{RGB}}$ and point cloud $P^{\mathrm{LiDAR}}$ information to generate a semantic mask map. In the MMPMM, each pixel encodes a mapping between the physical environment and communication performance. Static regions, such as buildings, exert stable effects on the communication channel. Dynamic regions, such as roads with moving vehicles or pedestrians, induce rapidly changing effects. By capturing these spatial and temporal variations, the MMPMM provides structured environmental knowledge to guide proactive beam prediction in V2I scenarios.

During the online beam prediction, the system uses both multi-model sensor data and MMPMM. Specifically, at time *t*, the BS data input is denoted as

(4) $$X_{t}=\{I_{\left(t-\Delta :t\right)}^{\mathrm{RGB}},P_{\left(t-\Delta :t\right)}^{\mathrm{LiDAR}},G_{\left(t-\Delta :t\right)}^{\mathrm{GPS}},K\},$$

where $G^{\mathrm{GPS}}$ denotes the position of the UE, (t−Δ:t) denotes the sequence of observations from time step t−Δ up to the current time *t*.

The goal is to predict the optimal beam index $b_{t}^{*}$ using multi-modal features

(5) $$b_{t}^{*}=f_{\Theta }(X_{t}),$$

where $f_{\Theta }$ is a deep neural network parameterized by Θ, trained to approximate the mapping between environmental knowledge and the corresponding optimal beam index under V2I scenarios.

The proactive beam prediction problem can thus be formulated as a supervised learning task. Given training data pairs $\left\{\left(X_{t},b_{t}^{*}\right)\right\}$, the model learns to minimize the expected prediction loss

(6) $$\min_{\Theta }E_{\left(X_{t},b_{t}^{*}\right)}[L\left(f_{\Theta }\left(X_{t}\right),b_{t}^{*}\right)],$$

where L(·) is typically the cross-entropy loss for classification tasks.

From an optimization perspective, the system aims to maximize the expected transmit power toward the optimal beam direction, which is equivalent to maximizing the expected achievable rate across all subcarriers. The optimization problem is formulated as

(7) $$\begin{array}{c} \max_{f_{t}\in F}E\left[R_{t}\left(f_{t}|X_{t}\right)\right]=E[\log_{2}\left(1+{\mathrm{SINR}}_{t}(f_{t}\right))],\\ \;\;\;\;\;\;\;\;s.t.f_{t}=f_{b_{t}},b_{t}=f_{\Theta }(X_{t}). \end{array}$$

## 3. Design of a Multi-Modal Prior Mask Map

Environment-aware communication (EAC) has emerged as a promising paradigm for future wireless networks [41]. Unlike conventional schemes that rely solely on instantaneous channel CSI, EAC does not passively wait for channel estimation. Instead, it observes the surrounding environment and infers potential variations in channel conditions, realizing the system to adapt its transmission strategy in advance. By exploiting geometric structures, material properties, and motion patterns in the scene, EAC builds an link between the physical environment and radio propagation.

**Figure 2:**
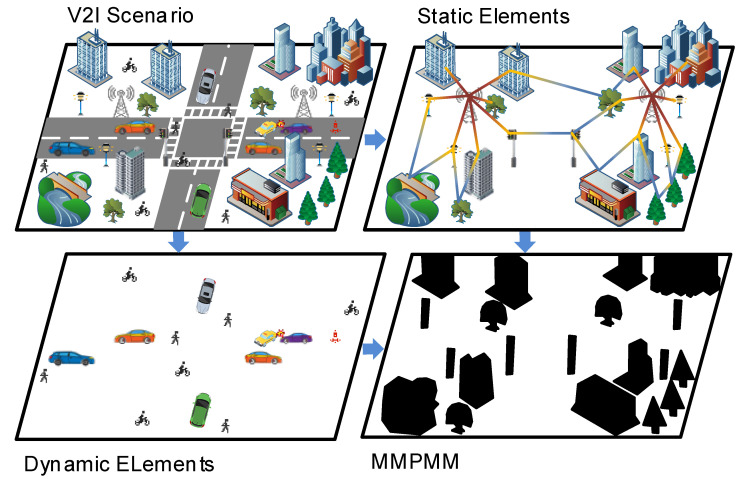
An illustration of the classification of elements in the V2I communication environment and the proposed MMPMM.

Based on this idea, we proposed the MMPMM framework, as illustrated in Figure 2. In V2I communication scenarios, the environment consists of two fundamental classes of elements, static structures and dynamic objects. These two classes affect mmWave propagation in different ways and therefore contribute unequally to beam prediction. The proposed MMPMM shares conceptual similarity with the local dynamic map (LDM) in intelligent transportation systems, where environmental elements are decomposed into static and dynamic components with distinct temporal characteristics. However, instead of maintaining a full multi-layer representation, MMPMM adopts a compact binary mask to encode motion-relevant regions, tailored for beam prediction in mmWave V2I communication. From a physical perspective, static structures such as buildings and road infrastructure determine the large-scale propagation geometry, including reflection surfaces and blockage boundaries, and thereby implicitly constrain the candidate beam space. In contrast, dynamic objects introduce time-varying blockage and are the main source of uncertainty in beam prediction. Accordingly, dynamic regions are assigned a value of 1 to highlight motion-related variations, while static regions are assigned 0. This design does not neglect the role of static structures; instead, it provides a task-oriented abstraction that explicitly emphasizes dynamic uncertainty while implicitly preserving the stable spatial constraints. Compared with more complex representations, the binary mask offers improved robustness and lower complexity.

Static structures determine the long-term geometry between the UE and the BS, including persistent reflection paths and large-scale attenuation patterns. These characteristics remain stable across consecutive frames. Therefore, static regions in images or LiDAR features carry redundant and repetitive information. When such patterns dominate the input feature maps, deep networks may overfit background textures instead of learning propagation related information. This reduces the model’s sensitivity to subtle but critical changes caused by moving objects, which are the actual drivers of short-term beam switching. Assigning a mask value of 0 to static regions suppresses these low-value patterns and forces the network to focus on inter-frame variations that truly affect beam dynamics.

In contrast, dynamic objects alter LOS blockage conditions, reflection and scattering paths, Doppler shifts, and near-field geometry relative to the BS. Highlighting dynamic regions with a mask value of 1 helps the model capture how moving vehicles or pedestrians modify available paths and how reflections evolve as objects transition across frames.

Importantly, MMPMM is constructed as an offline, site-specific database. Thus, it introduces no additional latency during online beam prediction and provides channel-relevant prior knowledge without incurring extra communication overhead. To maintain its validity, the map leverages the temporal stability of large-scale structures in V2I environments, where static elements evolve slowly while dynamic objects are transient and are not explicitly stored. The map is updated in an event-driven manner and is reconstructed only when noticeable structural changes occur, such as infrastructure modification or road layout updates. This avoids frequent recomputation while preserving the effectiveness of the offline prior. The site-specific property lies in the encoding of geometric priors rather than the learning model itself. The network is trained to extract generalizable features, while the mask map captures environment-dependent structural information. For new deployment scenarios, only the offline map needs to be reconstructed without retraining the model, enabling efficient adaptation across different environments.

**Figure 3:**
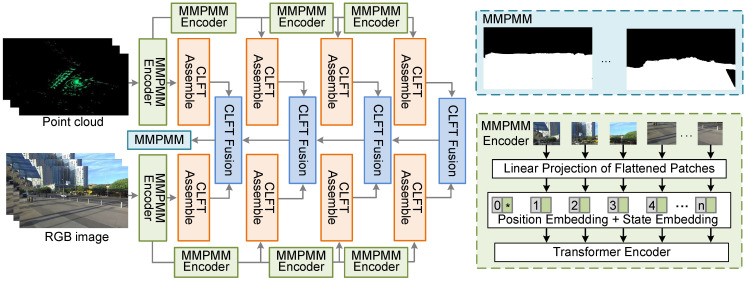
The diagram of the proposed MMPMM generation method based on point clouds and RGB images.

In this paper, we use RGB images and LiDAR point clouds as inputs to generate MMPMM. In network design, we refer to the application of vision Transformers (ViTs) in autonomous driving semantic segmentation, as shown in Figure 3. ViT divides the input image into fixed size patches, encodes each patch into a token, and successfully applies multi-head attention from NLP to computer vision tasks. Ref. [42] proposed the CLFT network for camera–LiDAR fusion in semantic segmentation. CLFT uses a assembly strategy in the encoder to gradually combine features from different layers into image like representations. In the decoder, a cross-fusion strategy integrates camera and LiDAR features progressively. This network has strong performance under challenging conditions such as rain and low light.

Inspired by the CLFT architecture, we propose an MMPMM extraction network that enhances environment awareness by incorporating the motion characteristics of semantic elements. While our design follows the general encoder–decoder structure of CLFT, the important contribution is the explicit encoding of both dynamic and static environmental state. This allows the network to capture motion information as well as stable structures that predefine the V2I communication environment.

In the encoder stage, we construct separate Transformer encoders for RGB images and LiDAR point clouds. Each encoder divides an input tensor $I^{\mathrm{RGB}}\in R^{H\times W\times C}$ and $P^{\mathrm{LiDAR}}\in R^{H\times W\times C}$ into a sequence of *N* non-overlapping patches of size p×p, where $N=\frac{H\cdot W}{p^{2}}$. Each patch $x_{i}$ is flattened into a vector and then linearly projected into a *D*-dimensional embedding token

(8) $$T_{i}=W_{\mathrm{proj}}\cdot \mathrm{flatten}\left(x_{i}\right)+b_{\mathrm{proj}},\;i=1,\ldots ,N,$$

where $W_{\mathrm{proj}}\in R^{D\times (p^{2}C)}$ is the projection matrix and $b_{\mathrm{proj}}\in R^{D}$ is the bias term.

The complete input sequence for the Transformer encoder is then constructed by prepending a learnable state classification token $T_{\mathrm{cls}}\in R^{D}$ to the patch tokens and adding a positional embedding $E_{\mathrm{pos}}\in R^{(N+1)\times D}$ to retain spatial information

(9) $$T=\left[T_{\mathrm{cls}},T_{1},T_{2},\ldots ,T_{N}\right]+E_{\mathrm{pos}},$$

This multi-layer Transformer architecture processes the sequence T, where shallow layers capture local texture features and deeper layers extract rich global semantic representations.

In the decoder stage, we employs a progressive assembly strategy to reconstruct image like feature maps from tokens extracted at different encoder layers. Let $T^{\left(l\right)}\in R^{(N+1)\times D}$ denote the output token sequence from the *l*th encoder layer. The state class token $T_{\mathrm{cls}}^{\left(l\right)}$ is first excluded, and the remaining *N* patch tokens $\left\{T_{1}^{\left(l\right)},\ldots ,T_{N}^{\left(l\right)}\right\}$ are reshaped into a 2D feature map $X^{\left(l\right)}\in R^{\frac{H}{s_{l}}\times \frac{W}{s_{l}}\times D}$ based on their original spatial positions, where $s_{l}$ is a layer-specific scaling factor. This feature map is then processed by a convolutional projection layer to unify the channel dimension

(10) $${\hat{X}}^{\left(l\right)}=\mathrm{ConProj}\left(X^{\left(l\right)}\right)\in R^{\frac{H}{s_{l}}\times \frac{W}{s_{l}}\times \hat{D}},$$

where $\hat{D}$ is the projected feature dimension. This process generates a multi-scale pyramid of feature representations, shallow features with smaller $s_{l}$ values are upsampled to higher resolutions, while deep features with larger $s_{l}$ values retain lower resolutions, thereby preserving a consistent spatial hierarchy.

The fusion strategy involves progressively integrating the RGB and LiDAR feature maps, ${\hat{X}}_{\mathrm{RGB}}^{\left(l\right)}$ and ${\hat{X}}_{\mathrm{LiDAR}}^{\left(l\right)}$, through a series of residual convolution units (RCUs). The fusion process at level *l* is defined as

(11) $$F^{\left(l\right)}=\mathrm{RCU}(F^{\left(l-1\right)}+{\hat{X}}_{\mathrm{RGB}}^{\left(l\right)}+{\hat{X}}_{\mathrm{LiDAR}}^{\left(l\right)}),$$

where $F^{\left(0\right)}$ is initialized as a zero tensor. This design enables the network to automatically learn optimal fusion weights through back-propagation, effectively embodying a hybrid of late fusion and cross-fusion principles. The final fused feature map is passed through a convolutional and up-sampling module to generate the high-resolution semantic mask $K\in R^{H\times W\times C_{s}}$, where $C_{s}$ is the number of state classes for the V2I scenario elements. The MMPMM generation model is implemented based on the CLFT framework [42]. The supervision labels are obtained from semantic segmentation results and further converted into binary masks, where dynamic regions are labeled as 1 and static regions as 0. The training objective is formulated as a binary classification problem with two categories, and the binary cross-entropy (BCE) loss is adopted. The model is trained in a supervised manner using mini-batch stochastic gradient descent.

## 4. mmWave V2I Proactive Beam Prediction Based on the Multi-Modal Prior Mask Map

**Figure 4:**
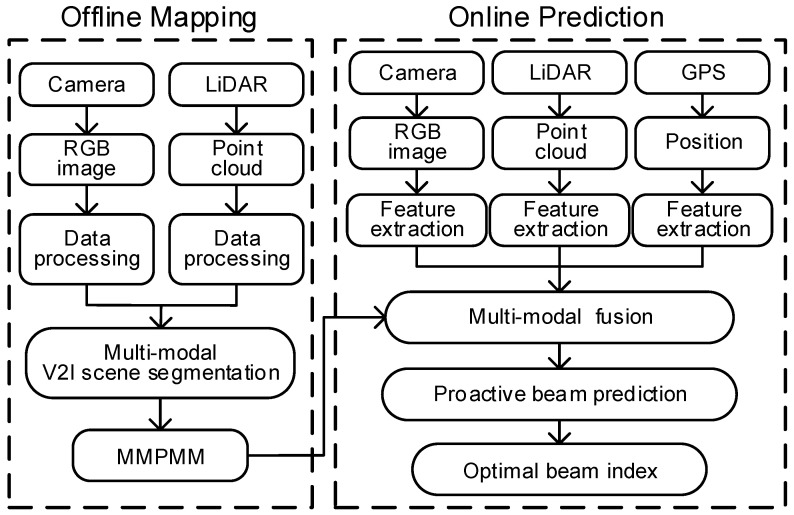
The flowchart of environment-aware proactive beam prediction based on MMPMM.

We propose an proactive beam prediction framework built on the MMPMM. It integrates real time multi-sensor environmental sensing with MMPMM, realizing BS to predict beam index with high accuracy and reliability, without pilot signals. The framework consists of two stages: offline mapping and online prediction. As shown in Figure 4, the offline stage collect rich environmental semantic priors, and the online stage leverages these priors for real time multi-modal beam prediction.

**Figure 5:**
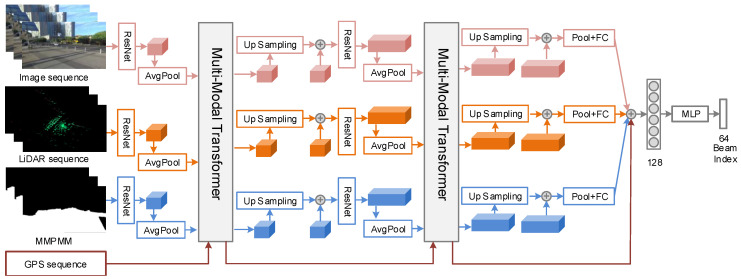
The framework of environment-aware proactive beam prediction, which uses image, LiDAR data, MMPMM, and GPS data as inputs and outputs the optimal beam index.

The offline map construction process is detailed in Section 3. During the online prediction stage, the framework of environment-aware proactive beam prediction, as shown in Figure 5, the BS real time collects RGB images, LiDAR point clouds, and GPS positions. In practice, these multi-modal inputs are first aligned in space and time to ensure consistency across all sensing streams. As this work focuses on beam prediction network and prior mask modeling, we do not study the alignment techniques in detail and assume that the inputs that are already aligned. A multi-modal feature extraction module is then applied to each sensor modality and the MMPMM, generating compact feature embeddings that describe texture, geometry, and position information. The extracted features are fed into a multi-modal fusion module, which combines the real-time sensor features with prior knowledge from the MMPMM. By incorporating the prior mask map into the fusion process, the model can identify environmentally stable structures that remain consistent over time and concentrate computational resources on dynamic areas that affect channel variation. Finally, the fused multi-modal tensor is used for beam prediction, where a neural network model learns to map the multi-modal features to the optimal beam index. The prediction module operates in a feed-forward manner, enabling the BS to anticipate future beam directions based on UE trajectory and environmental changes. Next, we present a detailed description of each network module.

### 4.1. Multi-Modal Data Processing and Feature Extraction

To enable effective mmWave proactive beam prediction between vehicles and infrastructure, it is essential to perform preprocessing and feature extraction on all multi-modal inputs. These steps reduce high-dimensional redundancy, capture environment semantics that relate to channel evolution, and generate compact and stable features for cross-modal fusion and prediction. Our system integrates four sensing modalities, RGB cameras, LiDAR, GPS, and the MMPMM, to provide a comprehensive and robust perception foundation for the beam prediction network.

(1) RGB Image Processing and Feature Extraction: RGB images captured by roadside cameras provide texture and structural information of the surrounding environment. However, in practical V2I scenarios, their quality is often degraded by illumination changes, shadows, and adverse weather. To address these issues, we apply a brightness enhancement model (MIRNet) to correct low-light regions and high-contrast areas, particularly in nighttime scenes [43]. This enhancement improves the visibility of UE contours and road boundaries, enabling the visual encoder to more reliably identify the UE. Each enhanced RGB frame is then resized and normalized before being passed into a ResNet-34 feature extraction backbone [44]. Feature extraction outputs a multi-scale image feature tensor $F_{\mathrm{RGB}}\in R^{C_{1}\times H_{1}\times W_{1}}$.
(2) LiDAR Data Processing and Feature Extraction: To leverage the geometric precision of LiDAR, the raw point clouds are first filtered and converted into a compact 2D representation. We remove static background structures using a moving average background subtraction,
  (12) $$P_{\mathrm{fg}}=P_{t}-\frac{1}{N}\sum_{i=t-N}^{t-1}P_{i},$$
  where $P_{i}$ is the point cloud at frame *i*. This step retains dynamic and semi-static objects, including vehicles and pedestrians, which are more relevant to beam variations.
  The filtered points are then projected into a bird’s eye view (BEV). For each grid cell, we encode height, intensity, and point density into the three channels of an image,
  (13) $$I_{\mathrm{BEV}}\left(x,y\right)=[\max\left(h_{x,y},\max\left(I_{x,y},\rho_{x,y}\right)\right)],$$
  where $h_{x,y}$ denotes the height statistic of the grid cell (x,y), $I_{x,y}$ represents the return intensity at that cell, and $\rho_{x,y}$ indicates the point density within the same location.
  This BEV representation preserves depth information and local geometry while reducing computational load. The resulting BEV image is then processed by a ResNet-18 feature extraction encoder to extract hierarchical geometric features, $F_{\mathrm{LiDAR}}\in R^{C_{2}\times H_{2}\times W_{2}}$. It is worth noting that the LiDAR preprocessing and the prior-map construction serve complementary roles. The former focuses on extracting dynamic cues for real-time prediction, while the latter encodes static environmental priors to constrain the beam search space.
(3) MMPMM Data Processing and Feature Extraction: The MMPMM is aligned with the spatial resolution of the input image to maintain pixel-level correspondence during feature extraction. When the mask is produced by an independent module, its size often differs from that of the target modality. To correct this mismatch, we resize the mask to match the spatial dimensions of the input image via nearest neighbor interpolation. Then, the mask map is processed by a ResNet-18 backbone to extract state-aware prior features, denoted as $F_{\mathrm{prior}}\in R^{C_{3}\times H_{3}\times W_{3}}$.

### 4.2. Multi-Modal Feature Fusion and Beam Prediction

We propose a multi-modal fusion framework that integrates visual, depth, positional, and scene motion state information into a unified representation for beam prediction. The Transformer-based architecture is designed to capture spatial structure, temporal evolution, and cross-modal interactions across RGB images, LiDAR measurements, GPS traces, and MMPMM masks. By jointly exploiting geometric information, semantic layout, and object dynamics, the framework predicts the optimal beam index under rapidly changing channel conditions.

The fusion process builds on hierarchical features extracted by the preceding modality specific encoders. The RGB stream uses a ResNet-34 backbone to capture texture, shape, and appearance information, while LiDAR, and the MMPMM are encoded using lightweight ResNet-18 networks that emphasize depth geometry, motion patterns, and static environmental structures. To ensure compatibility across modalities, all feature maps are projected into a shared embedding space and enhanced with positional encodings that preserve spatial layout and temporal order.

Following alignment, a Transformer encoder performs multi-level fusion through self-attention and cross-attention. Each modality is represented as a sequence of tokens sampled across time. The standard query key value formulation enables each modality to selectively attend to relevant information in the others, capturing inter-modal relationships such as the spatial consistency between camera views and LiDAR BEV geometry or the velocity correlation between LiDAR reflections and GPS-derived trajectories. Formally,

(14) $$\begin{array}{c} Z^{\left(l\right)}=\mathrm{MultiHeadAttn}\;\left(Q^{\left(l\right)},K^{\left(l\right)},V^{\left(l\right)}\right)+H^{\left(l-1\right)},\\ H^{\left(l\right)}=\mathrm{FFN}\;\left(Z^{\left(l\right)}\right)+Z^{\left(l\right)},\\ Q^{\left(l\right)}=W_{Q}^{\left(l\right)}H^{\left(l-1\right)},\\ K^{\left(l\right)}=W_{K}^{\left(l\right)}H^{\left(l-1\right)},\\ V^{\left(l\right)}=W_{V}^{\left(l\right)}H^{\left(l-1\right)}, \end{array}$$

where $H^{\left(l-1\right)}$ denotes the concatenated embeddings from all modalities at layer l−1, and $W_{Q}^{\left(l\right)}$, $W_{K}^{\left(l\right)}$, and $W_{V}^{\left(l\right)}$ are learnable projection matrices.

The MMPMM module enhances spatial semantic reasoning by highlighting regions that are physically relevant to mmWave propagation. Its mask annotations modulate the cross-modal attention weights, allowing the network to focus on critical propagation areas. Through two successive Transformer layers, the framework derives a compact and informative global representation. This representation is then passed to a multilayer perceptron with a softmax output to produce the probability distribution over the candidate beam indices. The predicted beam index $b_{t}^{*}$ is obtained via a maximum a posteriori decision

(15) $$b_{t}^{*}=\arg\max_{b\in B}P(b|f_{\mathrm{fusion}}),$$

where $f_{\mathrm{fusion}}$ denotes the fused multi-modal feature vector, $P(b|f_{\mathrm{fusion}})$ is the posterior probability generated by the softmax layer.

## 5. Simulation Results

### 5.1. Simulation Setup

To evaluate the performance of the proposed multi-modal proactive beam prediction framework, we conduct experiments on the DeepSense 6G dataset, a large-scale open dataset designed for AI driven wireless research and providing rich multi-modal sensing and communication data [45]. In this study, we use Scenes 32 to 34, which represent dynamic and complex V2I environments. Each scene offers synchronized RGB images, LiDAR point clouds, and GPS positions. The LiDAR and camera sensors are installed at the BS, while GPS measurements are collected at the UE. The wireless channel is described by a 64×1 power vector, corresponding to a predefined codebook of 64 beams. The adopted dataset is collected from real-world vehicular environments, which inherently capture stochastic variations such as sensing noise, mobility dynamics, and environmental changes.

At each time step *t*, the dataset provides a sequence of five samples, covering [t−4,…,t]. However, the UE GPS positions are available only for the first two steps, which simulates practical situations where GPS data may be missing. The learning objective is to predict the optimal beam index at time *t* by using past sensing information and partial position data.

We use three quantitative metrics for performance evaluation: the Top-*k* beam index prediction accuracy, the average power ratio (APR) [40], and the distance based accuracy (DBA) score [46]. The Top-*k* accuracy measures the proportion of test samples for which the ground truth optimal beam appears within the Top-*k* predicted indices.

We introduce the APR, which measures the ratio between the predicted receive power and the ground truth receive power. It reflects the impact of beam prediction errors at the system level, defined as

(16) $$\mathrm{APR}=\frac{1}{N}\sum_{n=1}^{N}(\frac{{\hat{y}}_{n}-y_{v}}{y_{n}-y_{v}}),$$

where $y_{v}$ denotes the noise floor of the scenario, ${\hat{y}}_{n}$ is the receive power of the predicted beam for sample *n*, and $y_{n}$ is the receive power of the ground truth beam. When k>1, we evaluate the receive power of the Top-*k* predicted beams and take the maximum among them as ${\hat{y}}_{n}$.

The DBA score offers a more informative assessment by evaluating how close the predicted indices are to the ground truth index within the codebook rather than treating all mispredictions equally. It is computed as

(17) $${\mathrm{DBA}}_{\mathrm{Score}}=\frac{1}{3}\sum_{m=1}^{3}Y_{m},$$

(18) $$Y_{m}=1-\frac{1}{N}\sum_{n=1}^{N}\min_{1\leq m^{\prime }\leq m}[\min\left(\frac{|{\hat{b}}_{\left(n,m^{\prime }\right)}-b_{n}|}{\gamma },1\right)],$$

where $b_{n}$ is the ground truth beam index of sample *n*, ${\hat{b}}_{\left(n,m^{\prime }\right)}$ is the $m^{\prime }$-th ranked predicted index, and γ=5 normalizes the maximum allowable index deviation. The DBA metric rewards models that place the correct beam close to the top of their ranked predictions, offering a balanced measure of both accuracy and index proximity.

The development dataset is split into training and validation sets using a random partition strategy at the sample level, with 90% of the data used for training and the remaining 10% for validation. Although the dataset is organized into multiple scenarios, these scenario labels are not used as strict boundaries for dataset partitioning; instead, they are leveraged for auxiliary analysis of performance under different environments. The batch size is set to 2 and the initial learning rate is $5\times 10^{-4}$. To stabilize the optimization, we use gradient accumulation with an accumulation step of 6. This approach allows us to reach an effective batch size beyond the GPU memory limit, giving a total effective batch size of 12. In addition, key architectural configurations are provided to improve reproducibility. The model takes a temporal sequence of T=5 frames as input. Multi-modal features are extracted using ResNet-34 for images and ResNet-18 for LiDAR and MMPMM inputs. The extracted features are processed through a multi-scale Transformer-based fusion architecture. Each Transformer module consists of 8 stacked layers with 4 attention heads, and the feed-forward expansion ratio is set to 4. Before being fed into the Transformer, spatial features are downsampled to a fixed grid of 8×8 anchors, ensuring a unified representation across modalities.The full set of experimental hyperparameters is summarized in Table 2.

### 5.2. Results and Discussion

We first evaluate the influence of different forms of MMPMM in the environment-aware proactive beam prediction task. The goal is to examine how the errors of MMPMM influence the performance of the proposed framework. Then, we compare the proposed MMPMM based beam prediction method with existing state of the art (SOTA) approaches to assess its advancement. Finally, we conduct simulations on degraded datasets to examine the methods under noisy conditions and validate the adaptability and robustness of the proposed approach. For comparative analysis, we include MMFF [47], TII [36], cross-modal feature enhancement (CMFE) [37], vision-position (VP) [38] and a baseline algorithm [44].

(1) MMFF Algorithm [45]: The MMFF algorithm integrates RGB images, depth maps, and sub-6 GHz CSI to enable proactive beamforming. It uses a multi-modal feature extraction and fusion module, together with adaptive weight learning, to capture the motion features of the vehicle.
(2) TII Algorithm [36]: The TII algorithm integrates RGB images, radar data, LiDAR data, and GPS information to enable proactive beam prediction. The method employs a four-layer Transformer encoder to learn the latent relations among feature tokens from different modalities and time steps. In addition, focal loss and exponential moving averaging are used to improve model performance under imbalanced data conditions.
(3) CMFE Algorithm [37]: The CMFE algorithm combines RGB images and radar data to achieve proactive beam prediction. It integrates multipath-like data augmentation (MLDA), cross-modal feature enhancement (CMFE), and an uncertainty-aware dynamic fusion mechanism to predict beam indices. The method also adjusts the weights of different modalities in real time to adapt to varying information density.
(4) VP Algorithm [38]: The VP algorithm fuses visual and positional data for proactive beam prediction. It consists of a CNN-based feature extractor and an MLP classifier.
(5) Baseline algorithm [31]: The baseline algorithm uses only GPS data to perform proactive beam prediction.

To evaluate the influence of the errors of MMPMM in a V2I environment, we designed four comparison settings. In the static-only configuration, all pixels in the mask map are set as static (value 0), whereas in the dynamic-only configuration, all pixels are set as dynamic (value 1). The proposed method uses the true static and dynamic partition for each scene, marking dynamic regions as 1 and static regions as 0. In the no-mask-map configuration, the network receives only the real-time sensory inputs without any prior map. All experiments employ identical network architectures and training settings to ensure a fair comparison.

**Figure 6:**
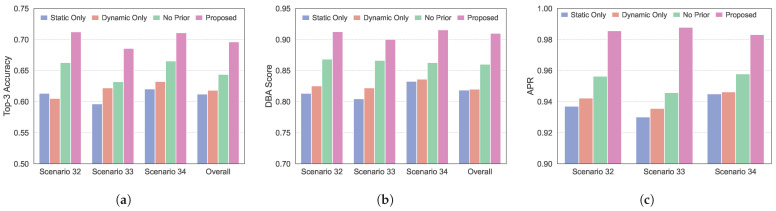
Impact of different forms of MMPMM on beam prediction performance in different scenarios. (**a**) Top-3 beam prediction accuracy. (**b**) Beam prediction DBA score. (**c**) The average power ratio.

Figure 6 shows the Top-3 accuracy, DBA score, and APR obtained by different forms of MMPMM in different scenarios. The results show that the proposed method achieves the highest performance among the compared methods across the evaluated scenarios and in all scenarios. As shown in Figure 6a, the proposed method achieves Top-3 accuracies of 0.7123, 0.6856, and 0.711 in Scenarios 32, 33, and 34, respectively. These values are more than 4% higher than without prior information. This improvement indicates that combining static structures with dynamic regions provides more effective constraints on the candidate beam directions and leads to higher accuracy and more stable predictions. In addition, the consistently highest Top-3 accuracy in every scenarios suggests a certain level of generalization across the evaluated scenarios.

Figure 6b,c presents the comparison results of the DBA score and the APR. The proposed method achieves the highest DBA score in all scenarios. Its DBA score is above 0.9 in the overall scenario, indicating that the model not only identifies the optimal beam but also ranks it above the suboptimal candidates with strong confidence. This improves the reliability of the beam selection decision. For the APR, the proposed method reaches values above 0.98 in all scenarios, showing that the received power of the predicted beam is close to that of the optimal beam and indicating that prediction errors introduce limited power degradation at the system level. In contrast, the APR of the other methods drops, which show that their beam index errors more easily lead to power degradation. The proposed method consistently maintains the smallest power loss in all scenarios, further demonstrating that the inclusion of MMPMM makes the model more sensitive to key environmental structures. It preserves stable beam-power performance even in complex or dynamic environments.

We also find that the proposed environment-aware proactive beam prediction framework continues to operate well in the static-only, the dynamic-only, and the no-prior setting. In all these cases, the Top-3 accuracy stays above 0.58, the DBA score remains above 0.8, and the APR exceeds 0.93. These results show that the framework is highly adaptable. Even when the MMPMM contains errors or when the prior map is missing, the framework is still able to maintain strong performance.

**Figure 7:**
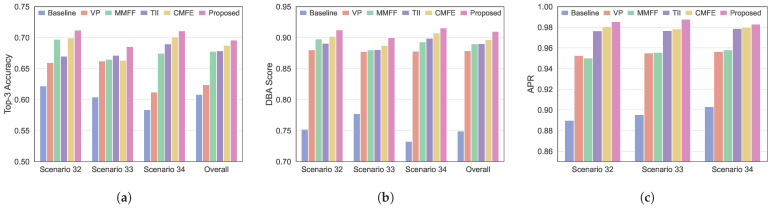
Comparison of the proposed method with classical methods in beam prediction performance across different scenarios. (**a**) Top-3 beam prediction accuracy. (**b**) Beam prediction DBA score. (**c**) The average power ratio.

To further evaluate the advantages of the MMPMM-based environment-aware beam prediction method, we compared it with several representative beam prediction approaches. It should be noted that these representative methods follow their original modality settings, which are not exactly the same. To address this, we refer to the above ablation results obtained under consistent input configurations to isolate the contribution of the proposed MMPMM. As shown in Figure 7a, the proposed method achieves the highest Top-3 accuracy in all scenarios. In scenarios 32 to 34, the Top-3 accuracy ranges from 0.68 to 0.71, whereas most competing methods remain below 0.68. A similar trend is observed in the DBA scores shown in Figure 7b. The proposed method consistently attains the best DBA performance in all scenarios, reaching 0.91 in overall scenario. Similarly, Figure 7c shows that the proposed method also achieves the highest APR. It is worth noting that all methods demonstrate relatively strong performance. The advantage stems from the environment-aware communication design, which captures environmental features through sensors and acquires early knowledge of the channel state, realizing proactive beam selection. In contrast, the baseline methods rely on GPS data only and lack the ability to predict complex environmental changes. Consequently, in V2I scenarios, they cannot reliably select the optimal beam direction.

To further evaluate the robustness of the proposed environment-aware MMPMM beam prediction method under adverse conditions, we introduced disturbances to both image and LiDAR inputs to simulate realistic sensor degradation. Specifically, for image data, Gaussian noise with a standard deviation of 0.05, normalized pixel range [0, 1], and motion blur with a kernel size of 7×7 pixels were applied to simulate heavy rain and fog conditions. For LiDAR point clouds, we randomly removed 30% of points for sparsification and added zero-mean Gaussian distance measurement noise with a standard deviation of 0.02 m to replicate reduced sensor resolution and interference. These combined perturbations allow for assessing the performance of all methods under conditions closer to real-world deployments.

**Figure 8:**
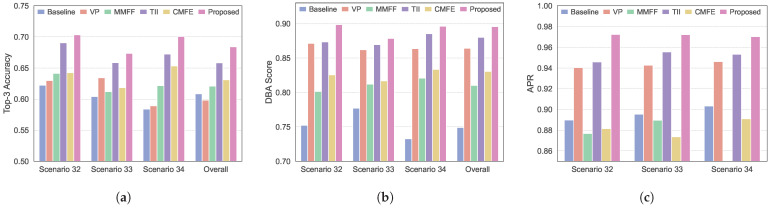
Comparison of the proposed method with classical methods in beam prediction performance under adverse conditions across different scenarios. (**a**) Top-3 beam prediction accuracy. (**b**) Beam prediction DBA score. (**c**) The average power ratio.

As shown in Figure 8, all methods exhibit performance degradation under adverse conditions. Nevertheless, the proposed environment-aware MMPMM-based proactive beam prediction method shows the smallest decline and consistently outperforms the other methods in terms of Top-3 accuracy, DBA score, and APR. This advantage stems from the MMPMM, which guides the model to focus on critical environmental features while constraining the candidate beam space, thereby maintaining reliable beam prediction even when sensor inputs are partially degraded. In addition, the baseline methods VP and TII exhibit less degradation than MMFF and CMFE, owing to their use of GPS data, which provides coarse spatial awareness under adverse conditions. Although GPS only information lacks detailed environmental representation, it still offers useful spatial priors that partially mitigate the impact of sensor noise.The overall robustness can be explained by the multi-modal design, where different sensing modalities provide complementary and partially redundant information, reducing sensitivity to individual sensor degradation. Meanwhile, the relative performance ranking among different methods remains largely unchanged across stochastic perturbations conditions, indicating that the observed trends are stable and not sensitive to specific perturbations.

Although the evaluations are conducted based on a real-world dataset, it is important to note that practical deployments may involve additional uncertainties, such as unseen environments, sensor calibration errors, and hardware heterogeneity, which may lead to performance variations compared to the reported results. Nevertheless, the proposed method incorporates multi-modal information and environment-aware modeling, which enhances its robustness to such variations. The consistent performance trends observed under different perturbation settings further suggest that the proposed approach has good potential to generalize to real-world scenarios.

Table 3 presents the computational complexity of different methods in terms of the number of parameters and FLOPs. The results show that the proposed method achieves a good balance between model size and computational cost. Specifically, MMFF offers high representational capacity but requires 74.04 M parameters and 56.85 G FLOPs, which is much higher than the other methods. TII and CMFE significantly reduce the FLOPs to approximately 11.4 G while maintaining a similar number of parameters 66–74 M. The VP method has the lowest computational cost among these advanced networks, with 33.02 M parameters and 7.14 G FLOPs, but this may come at the expense of reduced communication performance. In contrast, the baseline model is extremely lightweight, 0.03 M parameters and 0.05 M FLOPs, but offers very limited performance. Our proposed method achieves a moderate model size of 45.98 M parameters and 10.16 G FLOPs, showing a clear reduction in complexity compared with MMFF while maintaining sufficient capacity, which makes it more suitable for real-time or resource-limited applications. It is worth noting that the proposed MMPMM is constructed offline and does not introduce additional computational overhead during online inference. Therefore, the runtime latency mainly depends on the forward pass of the neural network, making the proposed method suitable for real-time or near-real-time deployment.

## 6. Conclusions

In this paper, we present a multi-modal perception-driven beam prediction framework based on the MMPMM, aimed at improving beam prediction accuracy and robustness under realistic and degraded sensing conditions. By jointly leveraging image and LiDAR features, the framework captures offline the semantic layout and geometric structures that influence mmWave propagation, which enables more reliable beam selection when online observations are incomplete or corrupted. Extensive experiments across multiple scenarios show that the proposed method consistently outperforms existing multi-modal prediction approaches in Top-3 accuracy, DBA score, and APR. Under the overall mixed-scenario setting, the proposed method achieves a Top-3 accuracy of 69.61% and a DBA score of 91.01%. The method maintains robust performance under adverse conditions where both image and LiDAR inputs are degraded by noise and sparsification, achieving a Top-3 accuracy of 68.41% and a DBA score of 89.55%. These results confirm the ability of MMPMM to reduce sensing uncertainty and preserve spatial consistency across modalities. Overall, this study demonstrates that combining multi-modal features with environment-aware knowledge modeling can significantly enhance beam prediction in complex V2I environments. The proposed framework offers a practical solution for mmWave communication systems that must operate reliably under adverse conditions. Despite the demonstrated effectiveness, the proposed approach has several limitations. The performance depends on the availability and quality of multi-modal sensory data and may degrade when severe sensor failures occur. In addition, the offline construction of the MMPMM introduces a site-specific dependency, requiring map reconstruction when significant environmental changes take place. Furthermore, the current framework does not explicitly model rapid dynamic variations in highly congested scenarios. These limitations will be addressed in future work.

## Figures and Tables

**Table 1:** Comparison of representative vision, LiDAR, and multi-modal beam prediction methods.

Method	Modality	Fusion Type	Prior Modeling	Environment Awareness	Prediction Paradigm
[22]	Vision	Implicit	×	✓	Proactive
[23]	Vision	Implicit	×	✓	Proactive
[26]	Vision	Implicit	×	✓	Reactive
[28]	LiDAR	Implicit	×	✓	Reactive
[29]	LiDAR	Implicit	×	✓	Proactive
[37]	Vision + Radar	Implicit	×	✓	Reactive
[38]	Vision + Position	Implicit	×	✓	Reactive
[32]	LiDAR + Position	Implicit	×	✓	Reactive
[35]	LiDAR + Radar	Implicit	×	✓	Reactive
[33]	Vision + LiDAR + Position	Implicit	×	✓	Reactive
[36]	Vision + LiDAR + Position	Implicit	×	✓	Proactive
**Proposed**	Vision LiDAR Position	Explicit guided	✓ MMPMM	✓	Proactive

**Table 2:** Hyperparameters for model training and architecture.

Parameter	Value
Backbone	
Image feature extractor	ResNet-34
LiDAR feature extractor	ResNet-18
MMPMM feature extractor	ResNet-18
Model Architecture	
Temporal window length *T*	5
Number of Transformer layers	8
Number of attention heads	4
Feed-forward expansion ratio	4
Spatial anchors	8×8
Training	
Total batch size	12
Epochs	100
Optimizer	AdamW
Learning rate	$5\times 10^{-4}$
Learning rate scheduler	Cyclic Cosine Decay

**Table 3:** Comparison of computational complexity across different methods.

Method	Parameters	FLOPs	Number of Modal
MMFF	74.04 M	56.85 G	3
TII	66.16 M	11.42 G	4
CMFE	74.03 M	11.37 G	2
VP	33.02 M	7.14 G	2
Baseline	0.03 M	0.05 M	1
Proposed	45.98 M	10.16 G	3

## Data Availability

The data are contained within the article.
